# Formate oxidation in the intestinal mucus layer enhances fitness of *Salmonella enterica* serovar Typhimurium

**DOI:** 10.1128/mbio.00921-23

**Published:** 2023-07-27

**Authors:** Maria G. Winter, Elizabeth R. Hughes, Matthew K. Muramatsu, Angel G. Jimenez, Rachael B. Chanin, Luisella Spiga, Caroline C. Gillis, Michael McClelland, Helene Andrews-Polymenis, Sebastian E. Winter

**Affiliations:** 1 Department of Internal Medicine, Division of Infectious Diseases, UC Davis School of Medicine, Davis, California, USA; 2 Department of Microbiology, UT Southwestern Medical Center, Dallas, Texas, USA; 3 Department of Pathology, Microbiology, and Immunology, Vanderbilt University Medical Center, Nashville, Tennessee, USA; 4 Department of Microbiology and Molecular Genetics, UC Irvine, Irvine, California, USA; 5 Department of Microbial Pathogenesis and Immunology, Texas A&M College of Medicine, College Station, Texas, USA; Fred Hutchinson Cancer Center, Seattle, Washington, USA; Nationwide Children's Hospital Center for Microbial Pathogenesis, Columbus, Ohio, USA

**Keywords:** *Salmonella*, metabolism, colitis

## Abstract

*Salmonella enterica* serovar Typhimurium induces intestinal inflammation to create a niche that fosters the outgrowth of the pathogen over the gut microbiota. Under inflammatory conditions, *Salmonella* utilizes terminal electron acceptors generated as byproducts of intestinal inflammation to generate cellular energy through respiration. However, the electron donating reactions in these electron transport chains are poorly understood. Here, we investigated how formate utilization through the respiratory formate dehydrogenase-N (FdnGHI) and formate dehydrogenase-O (FdoGHI) contribute to gut colonization of *Salmonella*. Both enzymes fulfilled redundant roles in enhancing fitness in a mouse model of *Salmonella*-induced colitis, and coupled to tetrathionate, nitrate, and oxygen respiration. The formic acid utilized by *Salmonella* during infection was generated by its own pyruvate-formate lyase as well as the gut microbiota. Transcription of formate dehydrogenases and pyruvate-formate lyase was significantly higher in bacteria residing in the mucus layer compared to the lumen. Furthermore, formate utilization conferred a more pronounced fitness advantage in the mucus, indicating that formate production and degradation occurred predominantly in the mucus layer. Our results provide new insights into how *Salmonella* adapts its energy metabolism to the local microenvironment in the gut.

**IMPORTANCE:**

Bacterial pathogens must not only evade immune responses but also adapt their metabolism to successfully colonize their host. The microenvironments encountered by enteric pathogens differ based on anatomical location, such as small versus large intestine, spatial stratification by host factors, such as mucus layer and antimicrobial peptides, and distinct commensal microbial communities that inhabit these microenvironments. Our understanding of how *Salmonella* populations adapt its metabolism to different environments in the gut is incomplete. In the current study, we discovered that *Salmonella* utilizes formate as an electron donor to support respiration, and that formate oxidation predominantly occurs in the mucus layer. Our experiments suggest that spatially distinct *Salmonella* populations in the mucus layer and the lumen differ in their energy metabolism. Our findings enhance our understanding of the spatial nature of microbial metabolism and may have implications for other enteric pathogens as well as commensal host-associated microbial communities.

## INTRODUCTION

The gut microbiota confers protection from infection with pathogenic organisms, a phenomenon termed colonization resistance (1, 2). Colonization resistance comprises both direct microbe-microbe interactions and indirect mechanisms that act via the host. Under homeostatic conditions, nutritional competition for complex polysaccharides is a key driver of the population structure (3, 4). As most small nutrient metabolites are absorbed in the small intestine, the primary carbon source for bacterial growth in the large intestine is complex polysaccharides, predominantly of dietary origin. The lack of energetically favorable carbon sources and nutrient competition with commensal bacteria might contribute to pathogen exclusion (5). Commensal gut microbes generate large quantities of short chain fatty acids, such as butyrate, propionate, and acetate. Short chain fatty acids directly inhibit growth of pathogenic *Salmonella enterica* serovar Typhimurium (*S*. Tm) and other Enterobacteriaceae family members (interference competition) (6, 7). Furthermore, release of antibacterial toxins by the gut microbiota suppresses colonization by pathogenic bacteria (8). In addition to microbe-microbe antagonism, gut commensal bacteria contribute to indirect, host-mediated mechanisms of colonization resistance. Colonocytes utilize butyrate as the primary carbon source through β-oxidation. The high oxygen demand of β-oxidation leads to a local depletion of oxygen, thus forming a suitable environment for the growth of obligate anaerobic fermenters (9, 10). Also, the host impedes pathogen colonization through release of antimicrobial peptides during infection (11, 12).

Enteric pathogens, such as *S*. Tm, have evolved to counter microbiota-based colonization resistance (13). For example, production of colicins or type six secretion systems allows *S*. Tm to suppress other Enterobacteriaceae family members (14, 15). In addition, the inflammatory response elicited by enteric pathogens leads to changes in the availability of metabolites. The niche created through inflammation is favorable to the growth of the pathogen (16, 17). Inflammatory reactive oxygen and nitrogen species are released locally to kill invading microbes that have breached the mucosal tissue. Leakage of these inflammatory radicals into the gut lumen results in off-target activity. Oxidation of thiosulfate leads to the production of tetrathionate (18), and peroxynitrite decays to nitrate (19). These molecules are used by *Salmonella* as terminal electron acceptors (18, 19). Furthermore, infection-driven changes in colonocyte metabolism lead to leakage of oxygen into the gut lumen (20). In the presence of terminal electron acceptors, *S*. Tm performs a respiratory central metabolism (21). This enables *S*. Tm to degrade toxic short chain fatty acids and bypass nutritional competition with obligate anaerobic fermenters (13, 21 - 23).

Terminal reductases are coupled via quinone pools to select membrane-bound dehydrogenases to form electron transport chains. In the murine gut, *S*. Tm utilizes host-derived L-lactate through its respiratory L-lactate dehydrogenase (LldD) (24 - 26). LldD couples to cytochrome bd oxidase-mediated oxygen respiration (24). It is unclear which respiratory dehydrogenases *S*. Tm uses during infection to donate electrons for nitrate and tetrathionate respiration. We thus sought to investigate dehydrogenases that participate in cellular respiration in *S*. Tm, with a focus on formate dehydrogenases.

Formate metabolism has been extensively studied in *Escherichia coli* (27). This organism produces two respiratory formate dehydrogenases, FDH-N and FDH-O. A third formate dehydrogenase, FDH-H is a component of the formate-hydrogen lyase complex involved in fermentation (28). FDH-N and FDH-O are each comprised of three subunits, FdnGHI and FdoGHI, forming symmetric trimers (FdnGHI)_3_ (29). Akin to *E. coli*, *S*. Tm utilizes formate as an electron donor *in vitro* (30). A comparison between the reference organisms *E. coli* K-12 MG1655 and *S*. Tm LT2 reveals that the subunits of the respective FDH-N (FdnGHI) and FDH-O (FdoGHI) enzymes exhibit significant amino acid sequence similarity (Fig. S1) and presumable the genes encoding these enzymes are orthologs. *S*. Tm also produces a fermentative FDH-H (28). In contrast to *E. coli*, little is known regarding the function of the respiratory formate dehydrogenase enzymes in *Salmonella*. In the current study, we investigated the physiological role of the respiratory formate dehydrogenases FDH-N and FDH-O in the context of *Salmonella* gut colonization.

## RESULTS

### Utilization of formate via respiratory FDHs enhances fitness in the presence of specific electron acceptors

**Fig 1:**
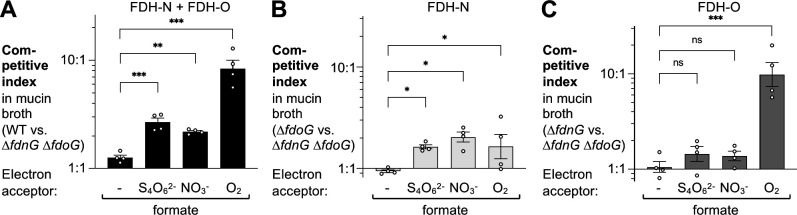
*S*. Tm utilizes formate as an electron donor for anaerobic respiration. Mucin broth, containing 0.5% porcine stomach type II mucin and 2 mM formate, was supplemented with 4 mM of tetrathionate (S_4_O_6_^2-^), or nitrate (NO_3_^-^), as indicated. Broth was inoculated with an equal mixture of the indicated strains and incubated anaerobically or under microaerobic conditions (1% O_2_) for 16 hours. The competitive index was calculated as the ratio of both strains recovered at the end of the experiment, corrected by the ratio in the inoculum. (A) Competitive fitness of the wild-type strain (AJB715) and a mutant lacking both FDH-N and FDH-O activity (SW1197). (B) To assess the contribution of FDH-N, the competitive fitness of a mutant lacking FDH-O (MW526) and a mutant lacking both FDH-N and FDH-O activity (SW1197) was determined. (C) Competitive fitness of a mutant lacking FDH-N (MW527) and a mutant lacking both FDH-N and FDH-O activity (SW1197) was determined assess the contribution of FDH-O. Bars represent the geometric mean ± geometric standard error. Each dot represents one biological replicate. *P < 0.05; **P < 0.01; ***P < 0.001; ns, not statistically significant.

Mutants of *S*. Tm that lack FDH-N activity have been characterized previously (31 - 33). For most of these mutants, genetic loci and genes were physically mapped using bacteriophage P22. Whole genome sequencing suggests that these mutations were likely pleiotropic, and mutations may have been in genes encoding L-seryl-tRNA^Sec^ selenium transferase, FDH-O, and/or FDH-H. Furthermore, *Salmonella* utilizes tetrathionate as a respiratory electron acceptor, while *E. coli* is unable to do so. We, therefore, sought to assess the physiological functions of the *S*. Tm respiratory FDHs, FDH-N, and FDH-O (Fig. 1). To address potential redundancy, we generated a mutant lacking the major subunits of FDH-N and FDH-O (*fdnG fdoG* mutant) and determined fitness in the presence of exogenous formate. To mimic the environment in the intestinal tract, we used broth containing porcine mucin (mucin broth). We inoculated mucin broth with an equal mixture of the *S*. Tm wild-type strain and an *fdnG fdoG* mutant. After 16 hours, we enumerated the abundance of each strain and calculated the competitive index as the ratio of the two strains in the media corrected by the ratio of the two strains in the inoculum. The wild-type strain was marked with a mutation in *phoN*, encoding an acidic phosphatase with no known role in virulence, to facilitate identification. Both strains were equally fit in the absence of exogenous electron acceptors during anaerobic growth (Fig. 1A). The wild-type strain outcompeted the *fdnG fdoG* mutant in the presence of the electron acceptors tetrathionate (anaerobic), nitrate (anaerobic), and oxygen (microaerobic). To evaluate the individual contribution of FDH-N and FDH-O, we repeated this experiment and determined the fitness of an *fdoG* mutant relative to the *fdnG fdoG* mutant (Fig. 1B) and an *fdnG* mutant relative to the *fdnG fdoG* mutant (Fig. 1C), respectively. The *fdoG* mutant outcompeted the double mutant in the presence of tetrathionate, nitrate, and oxygen, while the *fdnG* mutant only displayed a significant fitness advantage under microaerobic conditions. No fitness advantage was apparent for either single mutant over the double mutant in the absence of an exogenous electron acceptor (Fig. 1B and C). For genetic complementation, we introduced the *fdnG* and *fdoG* genes, under control of their native promoters, in a neutral locus in the chromosome (*phoN*); this complementation strategy restored fitness to wild-type levels (Fig. S2). Collectively, these results suggest that *S*. Tm FDH-O primarily couples formate oxidation to oxygen respiration, while the electrons liberated by the *S*. Tm FDH-N enzyme are donated to various electron acceptors under these experimental conditions.

### Respiratory formate dehydrogenases enhance fitness of *S*. Tm in the murine large intestine

**Fig 2:**
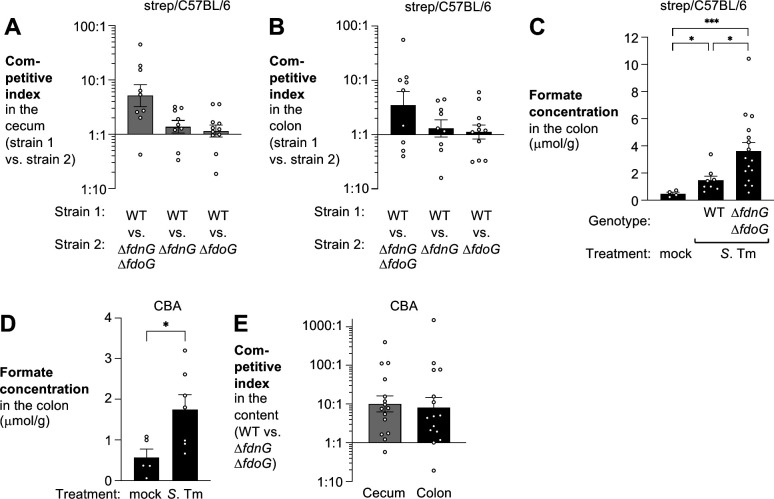
Respiratory formate dehydrogenases enhance *S*. Tm fitness in the murine gut. (A and B) C57BL/6 mice, pre-treated with streptomycin, were orally infected with an equal mixture of the wild-type (WT) strain (AJB715) and a mutant lacking respiratory formate dehydrogenase activity (SW1197), FDH-N activity (SW1195), or FDH-O activity (SW2182). Competitive fitness in the cecum content (A; gray bars) and the colon content (B; black bars) was determined 4 days after infection. Bars represent the geometric mean ± geometric standard deviation. (C) The concentration of formate in the colon content of mock-treated C57BL/6 mice and streptomycin pre-treated C57BL/6 mice infected with the *S*. Tm wild-type strain (IR715) or the respiratory formate dehydrogenase-deficient mutant (SW1197) was determined by gas chromatography-mass spectrometry. Bars represent the mean ± standard error. (D) Groups of CBA mice were orally infected with the wild-type strain. After 7 days, the concentration of formate in the colon content was quantitated by gas chromatography-mass spectrometry. Bars represent the mean ± standard error. (E) CBA mice were orally infected with an equal mixture of the wild-type (WT) strain (AJB715) and a mutant lacking respiratory formate dehydrogenase activity (SW1197). Competitive fitness in the cecum content (gray bar) and the colon content (black bar) was determined 7 days after infection. Bars represent the geometric mean ± geometric standard deviation. Each dot represents data obtained from one animal. *P < 0.05; ***P < 0.001.

We next investigated *S*. Tm formate metabolism in a murine model of *Salmonella*-induced colitis. Oral treatment of C57BL/6 mice with streptomycin increases susceptibility to *S*. Tm infection (34, 35). In this model, the inflammatory infiltrate in the intestinal tissue is dominated by neutrophils (35), akin to human infection with non-typhoidal *Salmonella* (36, 37). We infected groups of streptomycin treated mice with an equal mixture of the *S*. Tm wild-type strain and the *fdnG fdoG* mutant, the wild-type strain and a *fdnG* mutant, or the wild-type strain and a *fdoG* mutant and determined the abundance of each strain in the cecal and colon contents 4 days after infection (Fig. 2A and B). The wild-type strain was recovered in higher numbers than the *fdnG fdoG* double mutant, indicating that respiratory FDH activity enhances fitness of *S*. Tm during infection. The single mutants (*fdnG* mutant and *fdoG* mutant) were as fit as the wild-type strain (Fig. 2A and B), suggesting that FDH-N and FDH-O may fulfill redundant roles during *S*. Tm infection in this animal model.

We had previously shown that formate concentrations in the murine lumen increase in a mouse model of non-infectious colitis (38). To assess whether formate levels increase during *S*. Tm-induced colitis, we monitored formate concentrations in the cecal lumen by gas chromatography-mass spectrometry (Fig. 2C). Mice that were treated with streptomycin and infected with the *S*. Tm wild-type strain exhibited significantly elevated levels of formate (1.5 µmol/g) compared to mock-treated animals (0.48 µmol/g). The concentration of formate was further elevated (3.6 µmol/g) when we inactivated the ability of *S*. Tm to utilize formate through respiration (*fdnG fdoG* mutant), a finding that is consistent with consumption of formate by *S*. Tm (Fig. 2C).

Oral administration of streptomycin disturbs the composition of the gut microbiota (16, 39). We thus determined whether *S*. Tm to utilizes formate in the presence of an unperturbed microbiota. Unlike C57BL/6 mice, CBA mice survive infection with *S*. Tm and develop a neutrophilic inflammatory response in their large intestine between 7 and 10 days after infection (21). In the CBA mouse model, the formate concentration in the lumen of the colon rises significantly 7 days after *S*. Tm infection (Fig. 2D). Furthermore, FDH-N and FDH-O provide a fitness advantage in the cecum and colon contents (Fig. 2E). We conclude that utilization of formate through respiratory formate dehydrogenases enhances *S*. Tm fitness in the murine large intestine.

### Formate oxidation in the murine gut is coupled to several inflammation-derived electron acceptors

**Fig 3:**
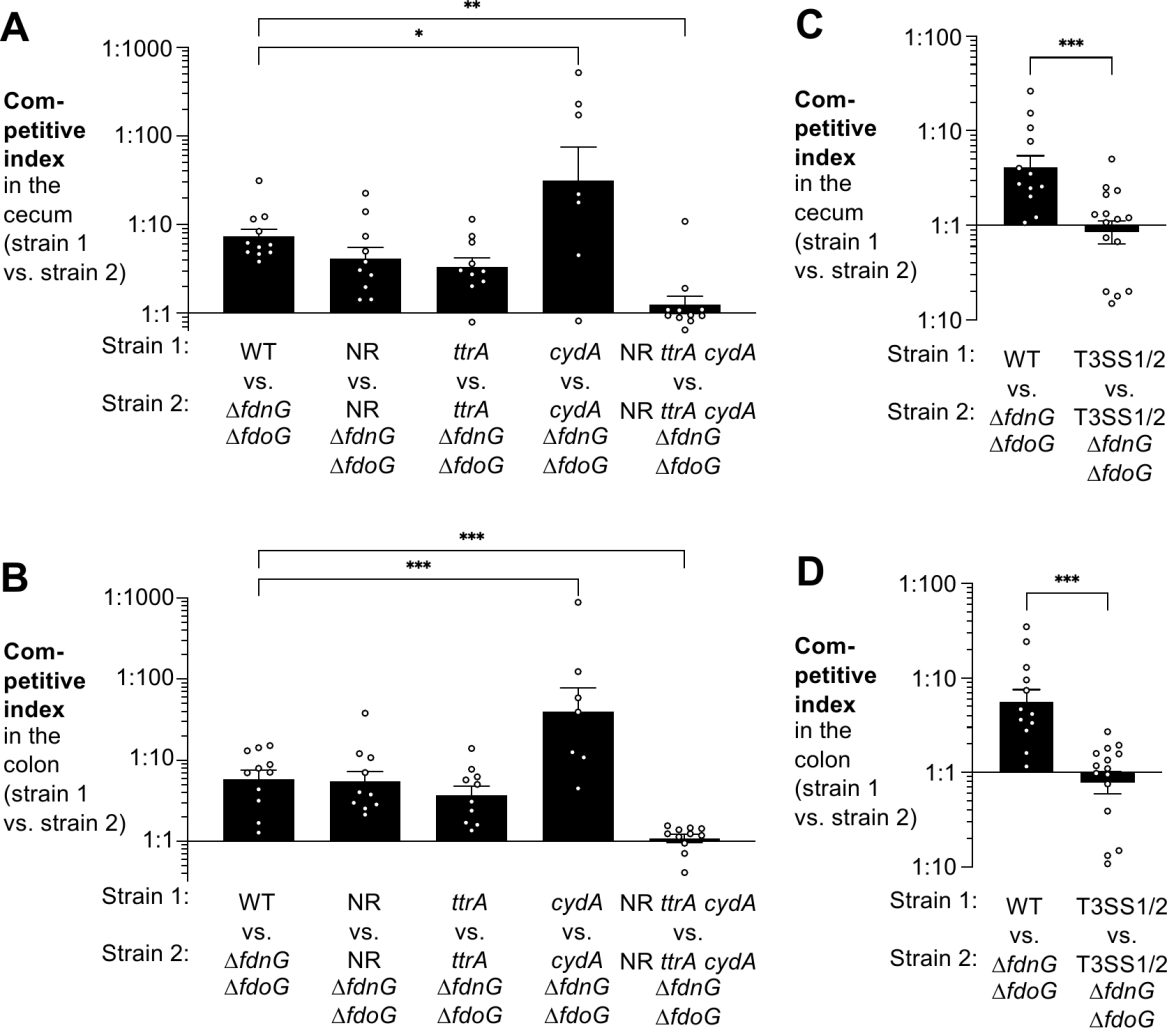
Respiratory formate dehydrogenases couple to multiple, redundant aerobic and anaerobic terminal reductases during *S*. Tm gut colonization. (A and B) C57BL/6 mice, pre-treated with streptomycin, were orally infected with an equal mixture of the *S*. Tm wild-type strain (IR715) and the respiratory formate dehydrogenase-deficient mutant (SW1197), or an equal mixture of the indicated strains lacking nitrate reductase (NR) (MW485 vs MW486), tetrathionate reductase (MW561 vs MW562), cytochrome bd-II oxidase (MW496 vs MW550), or all the aforementioned reductase activities (MW412 vs MW413). Competitive fitness in the cecum content (A) and the colon content (B) was determined 4 days after infection. (C and D) C57BL/6 mice, pre-treated with streptomycin, were orally infected with an equal mixture of the *S*. Tm wild-type strain (IR715) and the respiratory formate dehydrogenase-deficient mutant (SW1197) or a mixture of an avirulent strain (T3SS1/2; SW1401) and an avirulent strain lacking respiratory formate dehydrogenases (T3SS1/2 Δ*fdnG* Δ*fdoG*; SW1201). Competitive fitness in the cecum content (C) and the colon content (D) was determined 4 days after infection. Bars represent the geometric mean ± geometric standard error. Each dot represents one animal. *P < 0.05; **P < 0.01; ***P < 0.001.

We next sought to determine which electron acceptor(s) enable formate oxidation in the murine gut. We performed competitive colonization assay in strains lacking the three nitrate reductases (NR mutant), the tetrathionate reductase (*ttrA* mutant), and oxygen respiration (*cydA* mutant) (Fig. 3A and B). The growth advantage conferred by FDH-N and FDH-O was still observed in the absence of nitrate and tetrathionate respiration and was even more pronounced in the absence of cytochrome bd-II oxidase-mediated oxygen respiration. One explanation for these findings could be potential redundancy in the utilization of these different electron acceptors. Consistent with this idea, FDH-N and FDH-O ceased to provide a fitness advantage when we removed the ability to utilize nitrate respiration, tetrathionate reduction, and cytochrome bd-II oxidase-mediated oxygen respiration (Fig. 3A and B).

Nitrate and tetrathionate have been shown to be generated as byproducts of inflammatory reactive oxygen and nitrogen metabolism in the gut lumen (18, 19). Furthermore, oxygen availability in the gut lumen increases during inflammation (9, 20). We, therefore, tested whether inflammation is required for *S*. Tm formate utilization during infection. Inactivation of the two type III secretion system renders *S*. Tm non-invasive and unable to replicate in tissues (40). The fitness advantage conferred by formate oxidation was abolished in the absence of both type III secretions systems (T3SS1/2) (Fig. 3C and D), consistent with the idea that the availability of nitrate, tetrathionate, and oxygen increases when inflammation reshapes the metabolite landscape in the large intestine.

### *S*. Tm utilizes formate produced by its own metabolism as well as by the microbiota

**Fig 4:**
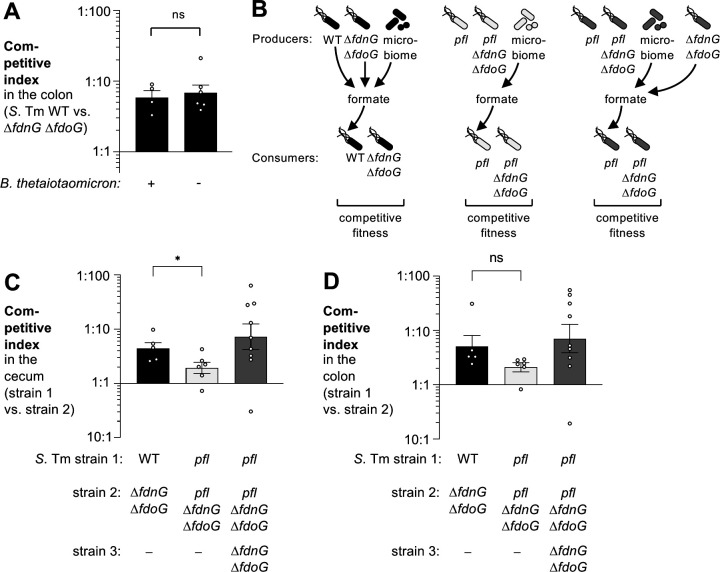
*S*. Tm pyruvate formate lyase activity contributes to the formate pool accessed by *S*. Tm. (A) Gnotobiotic Swiss Webster mice, either associated with *B. thetaiotaomicron* or mock treated, were infected with an equal mixture of the *S*. Tm wild-type strain (AJB715) and the Δ*fdnG* Δ*fdoG* mutant (SW1197). The competitive index of the colon content was determined 3 days after infection. (B-D) C57BL/6 mice, pre-treated with streptomycin, were orally infected with an equal mixture of the indicated strains. One group was infected with the wild-type strain (AJB715) and the Δ*fdnG* Δ*fdoG* mutant (SW1197) (black bars), one group was infected with a mutant lacking pyruvate formate lyase (*pfl*; MW519) and a mutant lacking formate dehydrogenase/pyruvate formate lyase activity (*pfl* Δ*fdnG* Δ*fdoG*; MW525) (light gray bars), and one group was infected with a mutant lacking pyruvate formate lyase (*pfl*; MW519), a mutant lacking formate dehydrogenase/pyruvate formate lyase activity (*pfl* Δ*fdnG* Δ*fdoG*; MW525), and a mutant lacking formate dehydrogenase (Δ*fdnG* Δ*fdoG* mutant; SW1197) (dark gray bars). (B) Schematic representation of the experimental design. (C) Competitive fitness of strains 1 and 2 in the cecum content. (D) Competitive fitness of strains 1 and 2 in the colon content. Bars represent the geometric mean ± geometric standard error. Each dot represents one animal. *P < 0.05; ns, not statistically significant.

In mouse models of non-infectious colitis, commensal *E. coli* utilizes primarily microbiota-derived formate (38) and we hypothesized that the formate utilized by *S*. Tm is also generated by the gut microbiota. We colonized gnotobiotic mice with *Bacteroides thetaiotaomicron*, an organism that supports cross feeding of formate to *E. coli* and to *Methanobrevibacter smithii* (41). We then infected groups of *B. thetaiotaomicron-*colonized and mock-treated mice with the *S*. Tm wild-type strain and the *fdoG fdnG* mutant, and assayed *Salmonella* colonization. Formate oxidation provided a similar fitness advantage in the presence and absence of *B. thetaiotaomicron* (Fig. 4A), suggesting that *S*. Tm either accesses dietary formate or produces formate as part of its central metabolism.

To better understand the origin of formate, we generated a *S*. Tm mutant (*pfl* mutant) lacking the three pyruvate-formate lyases (*pflB*, *pflD*, *pflF*) as well as the bifunctional pyruvate-formate lyase/2-ketobutyrate formate lyase (*tcdE*). *In vitro*, FDH-N and FDH-O provide a fitness advantage to wild-type cells under anaerobic conditions in the presence of tetrathionate, even when no exogenous formate is added (Fig. S3). This growth advantage is not observed in the *pfl* mutant background (*pfl* mutant vs *pfl fdnG fdoG* mutant), and it is rescued with the addition of formate to the growth media (Fig. S3). This implies that formate is released into the growth media as part of mixed acid fermentation, and then utilized by FDH-N and FDH-O.

To assess the contribution of different sources of formate in the streptomycin-treated mouse model, we analyzed the competitive fitness of a *pfl* mutant and a *pfl fdnG fdoG* mutant in the presence or absence of a strain that produces formate but that does not interfere with consumption (*fdnG fdoG* mutant). Each strain carries a different antibiotic resistance marker, enabling us to monitor bacterial colonization for each strain (Fig. 4B). The fitness advantage conferred by FDH-N and FDH-O was significantly decreased in the cecal content in the absence of pyruvate-formate lyase/2-ketobutyrate formate lyase (Fig. 4C). A similar trend was observed in the colon content (Fig. 4D), however, this difference was not statistically significant. Cross-feeding by the *fdnG fdoG* mutant to the *pfl*-deficient strains rescued the fitness phenotype conferred by formate oxidation in the *pfl* mutant background (Fig. 4C and D). We thus conclude that formate produced through pyruvate-formate lyase/2-ketobutyrate formate lyase activity is excreted by *S*. Tm, and then utilized by FDH-N and FDH-O on the periplasmic side of the inner membrane. *S*. Tm also likely accesses microbiota-derived formate since a modest growth advantage for formate oxidation remains in the *pfl* mutant background (Fig. 4C and D).

### Formate oxidation preferentially occurs in *Salmonella* cells residing in the mucus layer

**Fig 5:**
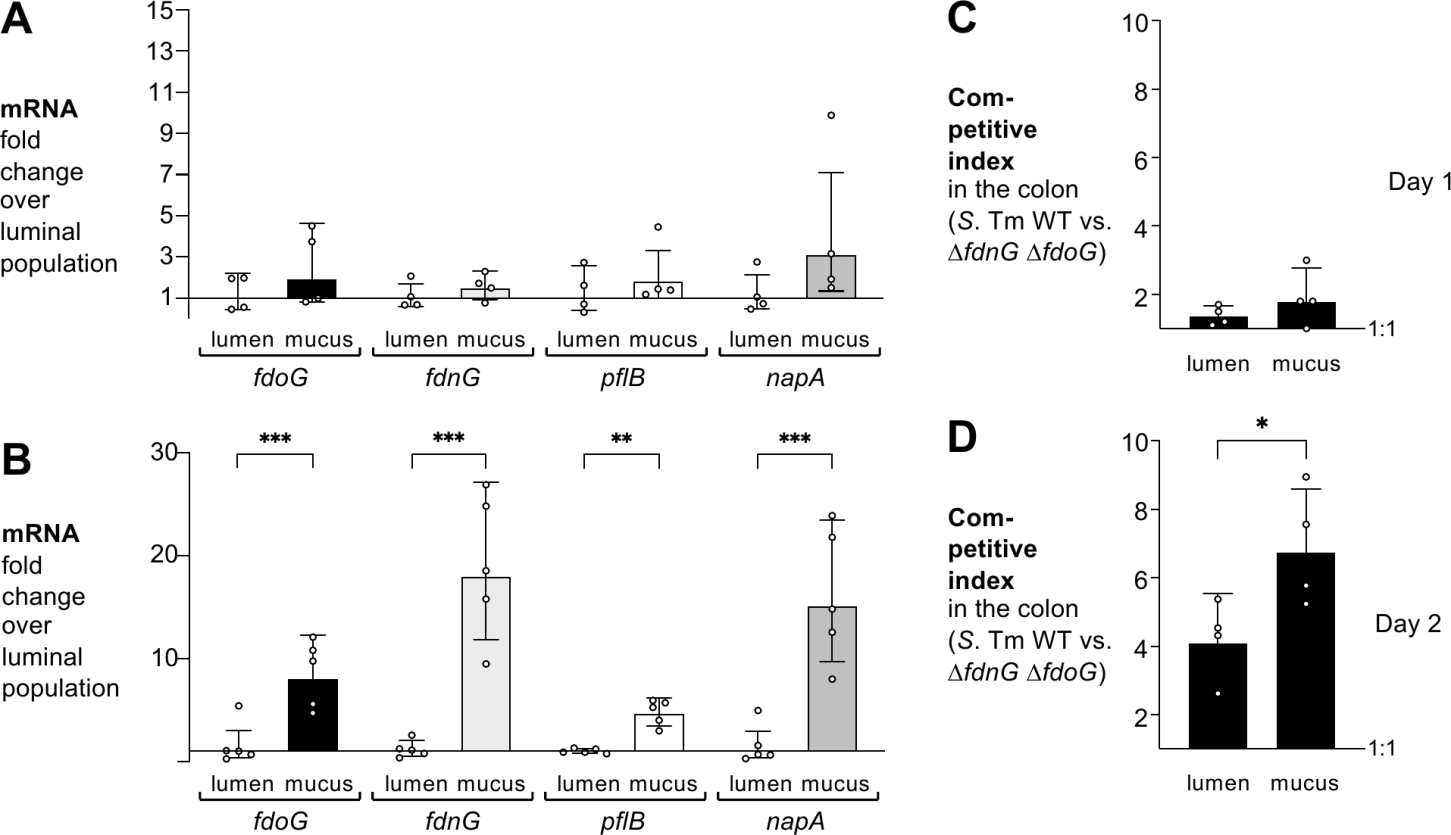
Formate oxidation occurs preferentially in the mucus layer. (A and B) Gnotobiotic Swiss Webster mice were orally infected with the *S*. Tm wild-type strain. One day (A) and 2 days (B) after infection, RNA was extracted from colonic mucus scrapings and lumenal content and mRNA levels of *fdoG* (black bars), *fdnG* (light gray bars), *pflB* (white bars), and *napA* (dark gray bars) determined. mRNA levels were normalized to *gmk*. (C and D) Gnotobiotic Swiss Webster mice were orally infected with an equal mixture of the *S*. Tm wild-type strain (AJB715) and the Δ*fdnG* Δ*fdoG* mutant (SW1197). The competitive index of the colon content and mucus was determined 1 day (A) and 2 days (B) after infection. Bars represent the geometric mean ± geometric standard deviation. Each dot represents one animal. *P < 0.05; **P < 0.01; ***P < 0.001

*Salmonella* colonizes both the lumenal space and the mucus layer. *Salmonella* subpopulations differ in their virulence gene expression (42), but it is unclear whether they differ in their metabolism. To address this question, we collected the luminal content and the mucus layer of *S*. Tm-infected gnotobiotic mice 1 and 2 days after infection, and assessed mRNA levels of *pflB*, *fdoG*, *fdnG,* and *napA* by RT-qPCR (Fig. 5A and B). No significant differences in mRNA levels of all these genes were found at the early time point, while mRNA levels were markedly increased in the mucus-associated subpopulation compared to the luminal population after 2 days (Fig. 5B).

To determine whether differences in formate utilization would result in altered fitness, we infected gnotobiotic mice with a mixture of the wild-type strain and the *fdnG fdoG* mutant and assessed fitness in the mucus layer and the luminal content 1 and 2 days after infection (Fig. 5C and D). Consistent with the transcriptional analysis, the wild-type strain and the formate oxidation deficient mutant were equally fit in the lumen and mucus layer at day 1. At day 2, the wild-type strain outcompeted the formate oxidation deficient mutant in both settings; however, the magnitude of the phenotype was significantly higher in bacteria residing in the mucus layer. These experiments suggest that formate consumption via FDH-N and FDH-O occurs preferentially in bacteria associated with the mucus layer.

## DISCUSSION

During gut colonization, *S*. Tm exhibits a versatile respiratory metabolism that depends on the increased availability of tetrathionate, nitrate, and oxygen (43, 44). Tetrathionate and nitrate are generated as byproducts of reactive nitrogen and oxygen metabolism, while oxygen influx during *S*. Tm infection is a consequence of inflammation-associated changes to host cell metabolism. The ability to reduce a vast number of exogenous electron acceptors is a property that sets *S*. Tm apart from most commensal bacteria and enables *S*. Tm to outcompete the gut microbiota (45). Here, we demonstrate that respiratory formate dehydrogenases are an important component of this inflammation adapted metabolism by donating electrons to the quinone pool and supporting respiration.

Respiratory formate dehydrogenases form electron transport chains with terminal reductases. The activities of these two enzymes are not only linked via the quinone pool but these protein complexes also physically interact and form supercomplexes (46). Since neither the enterobacterial nitrate reductases, cytochrome bd-II oxidase, tetrathionate reductase, nor the respiratory formate dehydrogenases are thought to exhibit true proton pump activity, proton translocation is likely achieved through two half redox loops in which proton consuming and generating reactions occur on separate sides of the cytoplasmic membrane (scalar chemistry) (47).

Respiration is not only more efficient in generating cellular energy, but it also enables *S*. Tm to access poorly fermentable carbon sources such as ethanolamine, 1,2 propanediol, and lactate (21, 23, 24, 48). These compounds are eventually converted to key molecules in the intermediary metabolism, such as acetyl-CoA and pyruvate, which can be used as building blocks for biosynthesis. In contrast, formate is oxidized to carbon dioxide, which is presumably lost to the environment. As such, the fitness advantage conferred by FDH-N and FDH-O is likely to be related to energy metabolism as an electron donor for respiration.

Respiratory formate dehydrogenases couple with specific terminal reductases according to substrate availability. For example, FDH-N couples with nitrate reductases, in particular NarGHI, when nitrate is present in the culture, while FDH-O is the dominant enzyme under microaerobic conditions *in vitro*. In *E. coli*, coupling is primarily due to gene expression (33). In mouse models of non-infectious colitis, commensal *E. coli* relies on FDH-N for optimal gut colonization. Unlike under *in vitro* conditions, FDH-N couples to cytochrome bd-II-mediated oxygen respiration in the murine inflamed gut (38). In the current study, we observed that *S*. Tm FDH-O was the predominant enzyme under microaerobic conditions *in vitro*, while formate oxidation via FDH-N was coupled to the reduction of nitrate, tetrathionate, and oxygen. In contrast to *E. coli*, *S*. Tm uses all three of these electron acceptors for formate oxidation in the murine gut. The cues and genetic factors that regulate expression of FDH-N and FDH-O in *Salmonella* are incompletely understood and require further investigation.

Formate utilization occurs in different animal models of *Salmonella* infection. A genetic screen for *Salmonella* colonization factors identified selenocysteine biosynthesis as a critical factor for chick colonization (49). The three FDHs are the only selenoproteins known to be produced by *E. coli* and *Salmonella* (50 - 52). Mutants unable to produce selenocysteine (*selD*) cannot metabolize formate *in vitro*. The colonization defect of *selD* mutants in chicks suggests that formate metabolism likely enhances fitness of *S*. Tm in this animal. Further work is needed to define whether the defect of the *selD* mutant in the chick gut is due to a lack of respiratory FDH-N and FDH-O, or fermentative FDH-H activity.

Formate is not only important for energy metabolism, but also serves as a cue to regulate expression of virulence factors. Formate induces production of the invasion-associated (SPI-1) type III secretion system in *S*. Tm by targeting *hilA* and *hilD*, which encode for key regulators of SPI-1 transcription (53, 54). Furthermore, formate induces virulence gene expression of *Shigella* during its intracellular stage (55).

Local availability of nutrients shapes microbial metabolism in the intestinal lumen and contribute to microhabitat formation. Both epithelial cells and infiltrating phagocytes contribute to nitrate production when homeostasis is perturbed (56, 57). During *S*. Tm-induced colitis, *S*. Tm utilizes primarily phagocyte-derived nitrate, while *E. coli* utilizes epithelial-derived nitrate during antibiotic-induced dysbiosis (57). These niches appear to be unique since an avirulent *S*. Tm strain is unable to access epithelial-derived nitrate in a setting of antibiotic-induced dysbiosis. Epithelial cells undergoing cell death release pyruvate into the gut lumen, thus providing *S*. Tm bacteria with an energetically valuable carbon source (58). In our study, pyruvate formate lyase was a contributor to the formate utilized by *S*. Tm. Host-derived pyruvate was metabolized in *S*. Tm by pyruvate formate lyase PflB (58). It is also conceivable that L-lactate, released by epithelial cells during *S*. Tm infection (24, 25), is taken up by *Salmonella* and converted to pyruvate. Our finding that formate utilization preferentially occurs in *S*. Tm bacteria associated with the mucus layer suggests the existence of a disease- and habitat-specific metabolism relying on formate oxidation via respiration. One limitation of our studies is that administration of streptomycin changes the composition of the gut microbiota, which may obfuscate the exact origin of formate during *S*. Tm infection of antibiotic-naïve animals.

Genetic evidence suggests that *Citrobacter rodentium*, a mouse pathogen that is closely associated with the colonic epithelium, uses FDH-N to colonize the murine intestinal tract (59). Our study on *Salmonella*, supported by this observation in *Citrobacter*, raises the possibility that formate utilization via respiratory formate dehydrogenases near the intestinal lining might be a common metabolic feature of enteric pathogens.

Curiously, formate and other short chain fatty acids are added to animal feed to prevent infection of livestock (60, 61). This notion suggests that not only the mere availability of any given metabolite determines microbial utilization, but also the local context matters. It is conceivable that formate, added in the diet, could change the composition of the gut microbiota. For example, Enterobacteriaceae use formate as an electron donor to support growth in the mammalian gut through respiration (38). *E. coli* respiration occurs in various settings in the murine gut, such as colitis and antibiotic treatment (38, 62, 63). The presence of Enterobacteriaceae interferes with *Salmonella* colonization in mice (15, 64 - 66). It is possible that administering subtherapeutic doses of antibiotics to livestock, commonly added to the feed to promote animal growth, enables respiration of Enterobacteriaceae in the gut. As such, a combination of dietary formate and subtherapeutic doses of antibiotics could favor colonization by commensal Enterobacteriaceae, which in turn could increase resistance to *Salmonella* colonization. Also, high levels of dietary formate could raise concentrations of this metabolite in different portions of the intestine, thus interfering with appropriate expression of T3SS-1 (53). Our study suggests that during *S*. Tm infection, the electron acceptors required for respiration emanate from the tissue, contributing to a local metabolic microenvironment suitable for *S*. Tm to perform formate oxidation.

## MATERIALS AND METHODS

### Bacterial strains and mutants

The bacterial strains used in this work are shown in Table 1. All *S*. Tm mutants were generated in IR715, a nalidixic acid resistant derivative of 14028S (67). Unless noted otherwise, we cultured *S*. Tm and *E. coli* strains aerobically in lysogeny broth (LB; 10 g/L tryptone, 5 g/L yeast extract, and 10 g/L sodium chloride) or on LB plates (10 g/L tryptone, 5 g/L yeast extract, 10 g/L sodium chloride, and 15 g/L agar) at 37°C. Antibiotics were added to LB and LB plates at the following final concentrations: carbeniciliin (Carb), 100 mg/L; nalidixic acid (Nal), 50 mg/L; kanamycin (Kan), 100 mg/L; and chloramphenicol (Cm), 15 mg/L (all Sigma-Aldrich, St. Louis, MO, USA). The *phoN* gene is a neutral locus in the *S*. Tm genome, and we used acidic phosphatase-deficient strains in all competitive fitness experiments (21). To detect acidic phosphatase (PhoN) activity, 5-bromo-4-chloro-3-indolyl phosphate (X-phos, Chem-Impex, Wood Dale, IL, USA) was added at a concentration of 100 mg/L to LB plates.

**TABLE 1:** Strains used in this study

Strain	Genotype	Source or reference
***Bacteroides thetaiotaomicron***		
ATCC29148 (VPI 5482)	Δ*tdk*	(68)
***E. coli***		
DH5⍺ λ*pir*	F^-^ *endA1 hsdR17* (r^-^m^+^) *supE44 thi-1 recA1 gyrA relA1* Δ(*lacZYA-argF*)*_U189_* f80*lacZ*ΔM15 λ*pir*	(69)
S17-1 λ*pir*	C600::RP4 2-(Tet::Mu) (Kan::Tn7) λ*pir recA1 thi pro hsdR* (r^-^ m^+^)	(70)
***Salmonella enterica* serovar Typhimurium**		
ATCC14028	Wild-type strain	ATCC
IR715	Fully virulent spontaneous nalidixic acid-resistant derivative of ATCC14028	(71)
2026	ATCC14028 Δ*fdnG*::Cm^R^	(72)
5179	ATCC14028 Δ*fdoG*::Kan^R^	(72)
SW1195	IR715 Δ*fdnG*::Cm^R^	This study
SW2182	IR715 Δ*fdoG*::Kan^R^	This study
SW1197	IR715 Δ*fdnG*::Cm^R^ Δ*fdoG*::Kan^R^	This study
AJB715	IR715 *phoN*::Kan^R^	(73)
SW284	IR715 Δ*phoN*::Cm^R^	(74)
MW526	IR715 Δ*fdoG*::Kan^R^*phoN*::Kan^R^	This study
MW527	IR715 Δ*fdnG*::Cm^R^*phoN*::Kan^R^	This study
RC141	IR715 Δ*fdoG*::Kan^R^ *phoN*::pRC29 (*fdoG*^+^)	This study
RC142	IR715 Δ*fdnG*::Kan^R^*phoN*::pRC30 (*fdnG*^+^)	This study
SPN487	IR715 Δ*invA* Δ*spiB*	(20)
SW1401	IR715 Δ*invA* Δ*spiB phoN*::Kan*^R^*	(21)
SW1201	IR715 Δ*invA* Δ*spiB* Δ*fdnG*::Cm^R^ Δ*fdoG*::Kan^R^	This study
SW661	IR715 *ttrA*::pSW171	(18)
MW561	IR715 *ttrA*::pSW171 *phoN*::Kan^R^	This study
MW562	IR715 *ttrA*::pSW171Δ*fdnG*::Cm^R^ Δ*fdoG*::Kan^R^	This study
CG40	IR715 Δ*narG* Δ*narZ* Δ*napA*	This study
MW485	IR715 Δ*narG* Δ*narZ* Δ*napA phoN*::Kan^R^	This study
MW486	IR715 Δ*narG* Δ*narZ* Δ*napA* Δ*fdnG*::Cm^R^ Δ*fdoG*::Kan^R^	This study
MW472	IR715 Δ*cydA*	This study
MW496	IR715 Δ*cydA phoN*::Kan^R^	This study
MW550	IR715 Δ*cydA* Δ*fdnG*::Cm^R^ Δ*fdoG*::Kan^R^	This study
MW559	IR715 Δ*narG* Δ*narZ* Δ*napA* Δ*cydA*	This study
MW409	IR715 Δ*narG* Δ*narZ* Δ*napA* Δ*cydA phoN*::Kan^R^	This study
MW411	IR715 Δ*napA* Δ*narZ* Δ*narG* Δ*cydA* Δ*fdnG*::Cm^R^ Δ*fdoG*::Kan^R^	This study
MW412	IR715 Δ*narG* Δ*narZ* Δ*napA* Δ*cydA ttrA*::pSW171 *phoN*::Kan^R^	This study
MW413	IR715 Δ*napA* Δ*narZ* Δ*narG* Δ*cydA ttrA*::pSW171 Δ*fdnG*::Cm^R^ Δ*fdoG*::Kan^R^	This study
MW364	IR715 Δ*pflF* Δ*pflD* Δ*tdcE*	This study
MW548	IR715 Δ*pflF* Δ*pflD* Δ*tdcE phoN*::Cm^R^	This study
MW519	IR715 Δ*pflF* Δ*pflD* Δ*tdcE phoN*::Cm^R^ *pflB*::pMW304	This study
MW557	IR715 Δ*pflF* Δ*pflD* Δ*tdcE* Δ*fdnG*::Cm^R^ Δ*fdoG*::Kan^R^	This study
MW525	IR715 Δ*pflF* Δ*pflD* Δ*tdcE* Δ*fdnG*::Cm^R^ Δ*fdoG*::Kan^R^*pflB*::pMW304	This study

The plasmids used in this study are listed in Table 2. New plasmids were generated using Gibson Assembly (New England Biolabs, Ipswich, MA, USA). DNA fragments were amplified using Q5 Hot Start High Fidelity DNA Polymerase (New England Biolabs) and *S*. Tm IR715 as a template. The primers used for mutagenesis are listed in Table 3. Suicide plasmids were propagated in *E. coli* DH5α λ*pir*. To generate pCG15, pCG25, and pCG27, the upstream and downstream regions of *narG*, *narZ*, and *napA* were PCR amplified and inserted into SphI-digested pRDH10 using the Gibson Assembly reaction. Similarly, for plasmids pMW301, pMW302, pMW303, DNA fragments comprising the upstream and downstream regions of *pflD*, *pflF*, and *tdcE* were introduced into SphI-digested pGP706. To generate pMW304, an internal fragment of the *S*. Tm *pflB* gene was amplified by PCR and cloned into the SphI site in pGP704 using a Gibson Assembly reaction. The promoter and coding sequence of *fdoG* and *fdnG* were PCR amplified and ligated into SphI-digested pSW327 to generate pRC29 and pRC30, respectively. The DNA sequence of inserts and key fragments was verified by Sanger sequencing.

**TABLE 2:** Plasmids used in this study

Plasmid	Relevant characteristics	Source or reference
pRDH10	*ori*R6K *mobRP4 cat sacRB*, Tet^r^	(67)
pSW327	*ori*R6K *mobRP4 bla ‘phoN’*	(21)
pGP704	*ori*R6K *mobRP4 bla*	(75)
pGP706	*ori*R6K *mobRP4 sacRB* Kan^R^	(24)
pCG15	Upstream and downstream regions of *S*. Tm *narG* in pRDH10	This study
pCG25	Upstream and downstream regions of *S*. Tm *narZ* in pRDH10	This study
pCG27	Upstream and downstream regions of *S*. Tm *napA* in pRDH10	This study
pCG124	Upstream and downstream regions of *S*. Tm *cydA* in pRDH10	(24)
pMW301	Upstream and downstream regions of *S*. Tm *pflD* in pGP706	This study
pMW302	Upstream and downstream regions of *S*. Tm *pflF* in pGP706	This study
pMW303	Upstream and downstream regions of *S*. Tm *tdcE* in pGP706	This study
pMW304	Internal fragment of *S*. Tm *pflB* in pGP704	This study
pRC29	*S*. Tm *fdoG* promoter and coding sequence in pSW327	This study
pRC30	*S*. Tm *fdnG* promoter and coding sequence in pSW327	This study

**TABLE 3:** Oligonucleotides used in this study

Target	Nucleotide sequence
*S*. Tm *narG*; deletion mutant	5′-GCCATCTCCTTGCATGACTGGCGGCGCTTTCAA-3′ 5′-CCGCCTTGTTGTGGTGGTACGTAAGATGAAG-3′ 5′-CACCACAACAAGGCGGACAGGGATGC-3′ 5′-CAAGGAATGGTGCATGCTGTATCTGGCGACCTTCG-3′
*S*. Tm *narZ*; deletion mutant	5′-GCCATCTCCTTGCATGAATTTCCCGACGTAAACATTC-3′ 5′-GTATTTTCATTTGTTTAAAGTAACGAAAGCG-3′ 5′-CTTTAAACAAATGAAAATACGCTCACAGGTTGG-3′ 5′-CAAGGAATGGTGCATGGGCAGCCGCTGATACACA-3′
*S*. Tm *napA*; deletion mutant	5′-GCCATCTCCTTGCATGTTGCCGCAGCCGGTACAG-3′ 5′-GAAAGCTAAATGTCCCGTACAGCGAAACC-3′ 5′-CGGGACATTTAGCTTTCATAAAGCTACGAC-3′ 5′-CAAGGAATGGTGCATGGACCTATTTTCTCGCACATTG-3′
*S*. Tm *pflD*; deletion mutant	5′-CTAGAGGTACCGCATGCTGGAAGGCAAGCTGGGTT-3′ 5′-GCTGTAGCCTTATCGGGCCTGTGAGC-3′ 5′-CCGATAAGGCTACAGCGCCTTCTTTG-3′ 5′-AGCTCGATATCGCATGCGTTTCAATAGCACAAGGAAC-3′
*S*. Tm *pflF*; deletion mutant	5′-CTAGAGGTACCGCATGCGCTAACCCGGCATGAATC-3′ 5′-ACTGAAACTCCTGATGGCGCTACGTTT-3′ 5′-CCATCAGGAGTTTCAGTTGGGTCATGATAATTATC-3′ 5′-AGCTCGATATCGCATGCGATCATGGCGACGGTACTG-3′
*S*. Tm *tdcE*; deletion mutant	5′-CTAGAGGTACCGCATGCTTTCACACAGCGTCAACAGTTC-3′ 5′-GCGATATGCGCGACGGATCTTCGCTG-3′ 5′-CCGTCGCGCATATCGCTGGTATCGATATTTAC CTTCATGAAAAATAATCTC-3′ 5′-AGCTCGATATCGCATGCATGGGGACTCGCAGCGGC-3′
*S*. Tm *pflB*; insertion mutation	5′-CTAGAGGTACCGCATGTCATTGGGTCCAGCTCGC-3′ 5′-AGCTCGATATCGCATGACACTCCGTATGAGGGTG-3′
*S*. Tm *fdnG*; complementation	5′-GCTTCTTCTAGAGGTACCGCATGCCCTTACGCCTTCTCGATG-3′ 5′-ATCGAGCTCGATATCGCATGCGCAAAAAAGTTATCGATTTTGC-3′
*S*. Tm *fdoG*; complementation	5′-GCTTCTTCTAGAGGTACCGCATGTTACACCTTTTCCACGTTC −3’ 5′-ATCGAGCTCGATATCGCATGGGACTTCTTCAGACAGTATATTG −3’
*S*. Tm *fdnG;* RT-qPCR	5′-CATAGCCGTTGTTCTCTTTC-3′ 5′-ACTATTGAAGATGAGCTGGAAC-3′
*S*. Tm *fdoG;* RT-qPCR	5′-ATAGCCAGTGAACAGACC-3′ 5′-TACTGACATTGCCTTCTT-3′
*S*. Tm *napA;* RT-qPCR	5′-TGAAAGAGAAAGGACCAGAAGCG-3′ 5′-TTGTTAGAGCGGAAACCAGCC-3′
*S*. Tm *pflB;* RT-qPCR	5′-TTTGCCTGCTTTCAGGTCAC-3′ 5′-GCTCAGGAAGCAATCCAGTG-3′
*S*. Tm *gmk;* RT-qPCR	5′-TTGGCAGGGAGGCGTTT-3′ 5′-GCGCGAAGTGCCGTAGTAAT-3′

For mutagenesis, plasmids were introduced into *E. coli* S17-1 λ*pir,* which served as the donor strain for conjugation into the appropriate *S*. Tm strains. A 1:1 mixture of the S17-1 λ*pir* donor and the *S*. Tm recipient was spread on an LB plate and incubated overnight at 37°C. After conjugation, bacteria were spread on LB plates supplemented with Cm (pRDH10 derivates), Carb (pGP704 and pSW327 derivatives), or Kan (pGP706 derivatives) to select for clones in which a single crossover event had occurred. Pure cultures of these strains were then grown in LB broth overnight at 37°C. Counterselection was performed by plating on sucrose plates (5% sucrose, 15 g/L agar, 8 g/L nutrient broth base; Thermo Fisher, Waltham, MA, USA) to recover clones with second crossover events. Clean, unmarked deletions of *narG*, *narZ*, and *napA* in IR715 were generated through repeated use of this mutagenesis strategy with plasmids pCD15, pCG25, and pCG27, respectively, giving rise to CG40. MW472 and MW559 were created by applying this mutagenesis strategy to IR715 and CG40, using pCG124. Similarly, unmarked deletions of *pflF*, *pflD*, and *tdcE* in IR715 were generated using plasmids pMW301, pMW302, and pMW303, giving rise to MW364. To generate the strains MW519, MW525, RC141, RC142, the plasmids pMW304, pRC29, and pRC30 were conjugated into MW548, MW557, SW2182, SW1195, respectively. Clones in which a single crossover (plasmid insertion) event had occurred were obtained by plating on LB agar containing Carb and Nal. Clean deletions and insertion mutations were confirmed by PCR.

Mutations marked with antibiotic resistance genes were introduced into different strain backgrounds by phage transduction using P22. Phage lysates were prepared by inoculating 4 mL of P22 broth (LB, 2 g/L glucose, E minimal media, ~10^8^ pfu/mL P22 HT-*int*) (76) with 1 mL of overnight culture. After 12 hours of aerobic incubation at 37°C, the culture was centrifuged, and the supernatant was sterilized with 0.2 mL chloroform. For transduction, 0.1 mL of serial 10-fold dilutions of phage lysate were mixed with 0.1 mL of recipient overnight culture, incubated at room temperature for 1 hour, and plated on LB agar plates containing the appropriate antibiotics. Single colonies were streaked on Evans Blue-Uranine (EBU) agar plates (10 g/L tryptone, 5 g/L yeast extract, 5 g/L NaCl, 2.5 g/L glucose, 15 g/L agar, 5 g/L K_2_HPO_4_, 12.5 mg/L Evans Blue, and 25 mg fluorescein sodium salt) (77). White colonies were evaluated for phage resistance using a cross streak with P22 H5. Single colonies were obtained on selective agar plates. The Δ*fdnG*::Cm^R^ and Δ*fdoG*::Kan^R^ mutations from strains 2026 and 5179, respectively, were introduced into IR715, SW1195, SPN487, CG40, MW472, MW559, and MW364 to create SW1195, SW2182, SW1197, SW1201, MW486, MW550, MW411, and MW557, respectively. The *phoN*::Kan^R^ and Δ*phoN*::Cm^R^ mutation from AJB715 and SW284 was transduced into CG40, MW559, and MW364 to generate MW485, MW409, and MW548, respectively. Similarly, the *ttrA*::pSW171 mutation from SW661 was introduced into AJB715, SW1197, MW409, MW411 to create MW561, MW562, MW412, MW413, respectively.

### *In vitro* growth experiments

Competitive growth experiments were performed as described in (18, 21). Briefly, raw porcine stomach type II mucin (Sigma-Aldrich) was suspended in 70% (vol/vol) ethanol, incubated at 65°C for 2 hours, and then incubated at room temperature overnight. The ethanol was evaporated in a vacuum centrifuge with mild heat. The sterilized mucin preparation was then resuspended in no-carbon E medium (0.2 g/L MgSO_4_ heptahydrate, 3.9 g/L KH_2_PO_4_, 5.0 g/L anhydrous K_2_HPO_4_, and 3.5 g/L NaNH_4_HPO_4_ tetrahydrate; Sigma-Aldrich) (78, 79). The final concentration of mucin was 0.5% (wt/vol). Sodium formate, sodium nitrate, and potassium tetrathionate (all Sigma-Aldrich) were dissolved in sterile water, filter-sterilized, and added at a final concentration of 2 mM (sodium formate) or 4 mM each (sodium nitrate, potassium tetrathionate), as indicated. For anaerobic growth experiments, the media was pre-incubated overnight in an anaerobic chamber (Bactron EZ; Sheldon Manufacturing, Cornelius, OR, USA). The atmosphere in the anaerobic chamber was composed of 5% carbon dioxide, 5% hydrogen, and 90% nitrogen. The absence of oxygen was confirmed on a routine basis (Oxoid, Basingstoke, Hampshire, United Kingdom). To prepare the inoculum, bacterial strains were pre-cultured in LB overnight under aerobic conditions at 37°C. Two milliliters of mucin broth was inoculated with 1 × 10^3^ CFU/mL of each strain incubated in an anaerobic chamber or a hypoxic chamber (1% oxygen, 99% nitrogen; Coy Lab Products, Grass Lake, MI, USA) at 37°C for 16 hours. Serial dilutions of the inoculum and the final culture were spread on LB plates supplemented with X-phos. The competitive index was calculated by correcting the ratio of the two strains of interest in the final culture by the corresponding ratio in the inoculum.

### Mouse experiments

All experiments were conducted in accordance with the policies of the Institutional Animal Care and Use Committees at UT Southwestern and UC Davis. Conventional C57BL/6 and CBA mice were originally obtained from The Jackson Laboratory. Animals were subsequently bred at UT Southwestern and UC Davis in barrier facilities under specific pathogen-free conditions. Germ-free Swiss Webster mice were maintained in plastic gnotobiotic isolators. All rooms were on a 12 hours light/dark cycle. Animals consumed food and water *ad libitum* throughout the experiment. Both male and female mice, aged 8–10 weeks, were used for experiments. We strived for equal representation of both sexes in each treatment group. No overt sex-specific differences were noted.

Naïve CBA mice were infected with 1 × 10^9^ CFU of *S*. Tm by gavage or mock treated with LB broth. C57BL/6 mice received 20 mg streptomycin sulfate (VWR) in water by gavage (35). Three days later, we administered 1 × 10^9^ CFU for single strain infection experiments, 5 × 10^8^ CFU of each *S.* Tm strain for a total of 1 × 10^9^ CFU for competitive infection experiments, or 3.3 × 10^8^ CFU of each *S.* Tm strain for a total of 1 × 10^9^ CFU for infection experiments involving three strains. Mice were euthanized at the indicated time points. Cecal and colonic tissues were flash frozen in liquid nitrogen and stored at −80°C. Cecal and colonic contents were dispersed in ice-cold, sterile phosphate buffered saline (PBS), the weight determined, and serial dilutions plated on LB plates supplemented with Nal and X-phos. The competitive index was calculated by dividing the ratio of the two strains of interest in the intestinal content by the corresponding ratio in the inoculum.

For the experiment shown in Fig. 4A, gnotobiotic Swiss Webster mice received 1 × 10^9^ CFU of *B. thetaiotaomicron* in 0.1 mL PBS. After 7 days, both groups were infected with 5 × 10^4^ CFU of each *S.* Tm strain for a total of 1 × 10^5^ CFU. The competitive fitness was determined as described above. For the experiment shown in Fig. 5, gnotobiotic Swiss Webster mice were either infected with 1 × 10^5^ CFU of the *S*. Tm wild-type strain or a mixture of 5 × 10^4^ CFU of each *S.* Tm strain, as indicated. The mucus layer was carefully removed by scraping with a spatula. The competitive fitness was determined as described above.

### Bacterial gene expression

Bacterial RNA was extracted from feces or mucus scrapes using the TRI reagent method (Molecular Research Center, Sunnyvale, CA, USA). Samples were initially homogenized in TRI reagent in a Mini BeadBeater (Biospec Products, Bartlesville, OK, USA). cDNA was generated using random hexamers and other TaqMan Reverse Transcription Reagents (Thermo Fisher, Waltham, MA, USA). To assess DNA contamination in the RNA preparation, a mock-RT-PCR, lacking reverse transcriptase, was performed for each sample and target gene. SYBR green-based real-time PCR was performed using the primers listed in Table 3 on a QuantStudio 6 Flex instrument (Thermo Fischer). Data were analyzed using the comparative Ct method. Gene expression was normalized to *S*. Tm *gmk* mRNA levels. Each mucus scrap sample was compared to the luminal sample from the same mouse.

### Quantitation of formate by GC/MS

GC/MS analysis was performed as described in reference (38). Briefly, luminal content from the cecum and colon was placed into ice-cold, sterile PBS and carefully dispersed by vortexing. The suspension was centrifuged at 20,000 g for 15 minutes at 4°C and the supernatant stored at −80°C. This procedure does not lyse representative gut commensal bacteria and thus likely reflects mostly extracellular metabolites (38). Formate was extracted from acidified samples with ethyl acetate. Dried extracts were derivatized with *N*-tert-butyldimethylsilyl-*N*-methyltrifluoroacetamide with 1% *t*-BDMCS (Cerilliant, Round Rock, TX, USA) at 80°C for 1 hour. Derivatized samples were analyzed by GC/MS (TQ8040; Shimadzu, Pleasanton, CA, USA). The injection temperature was 250°C, the injection split ratio was 1:100 with a volume of 1 µL. The oven temperature started at 30°C for 4 minutes, increasing to 230°C at 10°C per minute and to 330°C at 20°C per minute with a final hold at this temperature for 30 seconds. Helium flow rate was set to a constant linear velocity of 50 cm/s. The column used was a 30 m × 0.25 mm × 0.25 µm Rtx-5Sil MS (Shimadzu). The interface temperature was 300°C, the electron impact ion source temperature 200°C, with an ionization voltage of 70 V and a current of 150 μA. Selected ion monitoring with an event time of 50 ms was performed. The target and reference (qualifier) ions for formate were m/z = 103 and m/z = 75, respectively. The target and reference ions for deuterated formate were m/z = 104 and m/z = 76. Both internal and external standards were used to quantitate formate concentrations.

### Statistical analysis

Data were analyzed and processed in Microsoft Excel and GraphPad Prism v.9. All raw data were transformed with the natural logarithm prior to statistical analysis. Animals that were euthanized for health reasons prior to the end of the experiment were excluded from analysis. Similarly, mice that were insufficiently colonized (<10 colonies in 100 µL of undiluted sample) in competitive infection experiments were excluded from analysis. To determine statistical differences between groups of mice or treatment regimens *in vitro*, a two-tailed, unpaired Student’s *t*-test was applied to the logarithmically transformed data. For formate measurements by GC/MS, an unpaired Student’s *t*-test was used. *P* values less than 0.05 were considered significant.

## SUPPLEMENTAL MATERIAL

Supplemental figures mbio.00921-23-s0001.pdf Fig. S1-S3.

ASM does not own the copyrights to Supplemental Material that may be linked to, or accessed through, an article. The authors have granted ASM a non-exclusive, world-wide license to publish the Supplemental Material files. Please contact the corresponding author directly for reuse.
