# Supplementary material for: Formate oxidation in the intestinal mucus layer enhances fitness of Salmonella enterica serovar Typhimurium
Source: mBio. 2023 Jul 27;14(4):e00921-23. doi: 10.1128/mbio.00921-23 (PMC10470504; doi:10.1128/mbio.00921-23)
Supplement: Supplemental figures — Fig. S1-S3. [file mbio.00921-23-s0001.pdf]

A

MG1655 vs. LT2		MG1655 vs. LT2		LT2	
FdnG (α-subunit):	93.4%	FdoG (α-subunit):	94.0%	FdnG vs FdoG:	79.3%
FdnH (β-subunit):	93.2%	FdoH (β-subunit):	93.3%	FdnH vs FdoH:	74.2%
FdhI (γ-subunit):	98.2%	FdoI (γ-subunit):	96.2%	FdnI vs FdoI:	48.6%

B

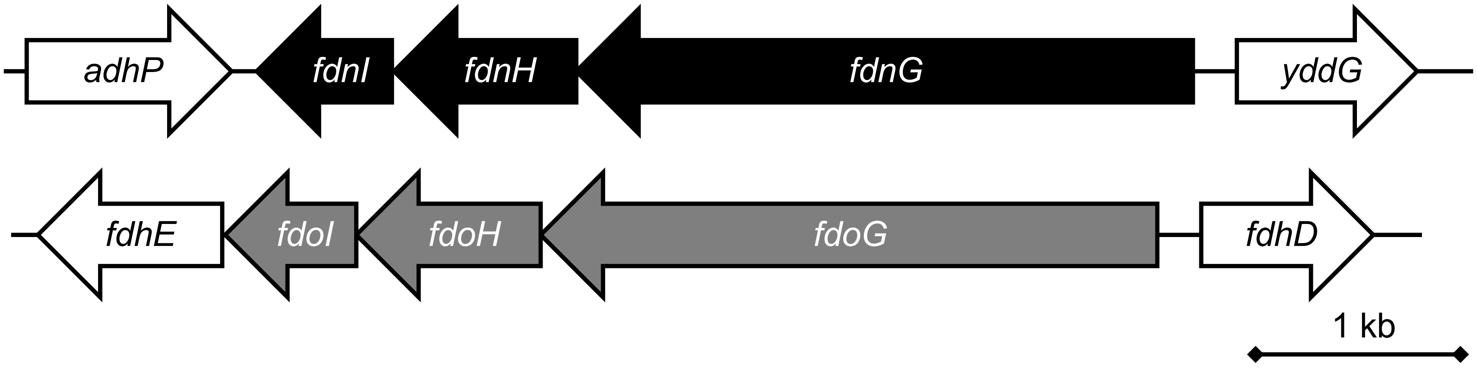

Figure S1. Sequence analysis of respiratory formate dehydrogenase genes and proteins in *S*. Tm.

(A) Sequence identity of the FDH-N and FDH-O proteins in *E. coli* MG1655 and *S*. Tm LT2. Sequence alignment was performed using blastp (BLOSUM62)

(B) Schematic representation of the *fdn* and *fdo* loci in *S*. Tm.

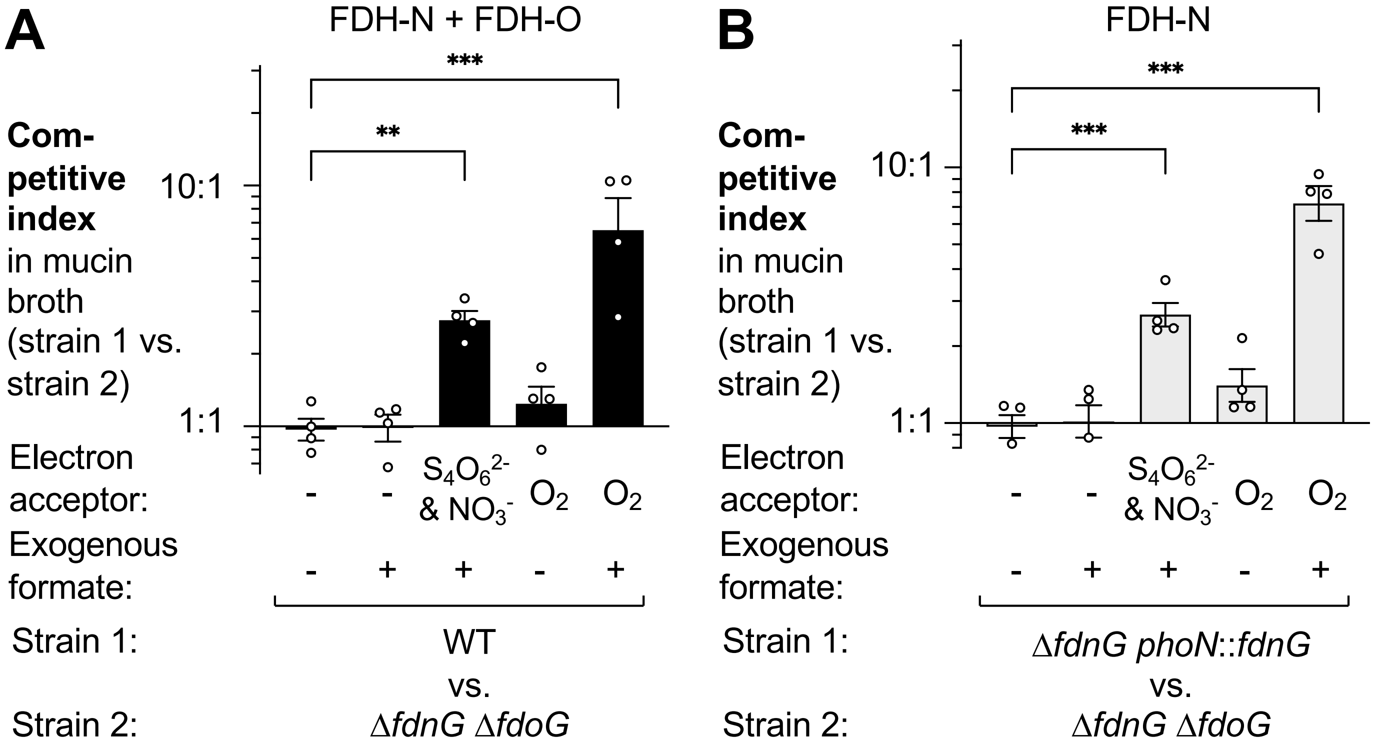

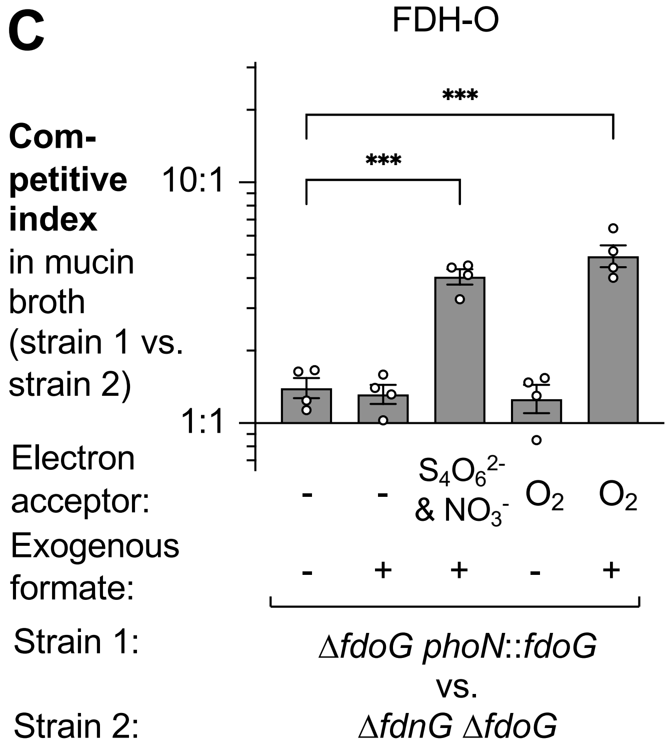

Figure S2. Genetic complementation of formate dehydrogenase-deficient strains. Related to Fig. 1

Mucin broth was supplemented with 4 mM of formate, tetrathionate ($S_4O_6^{2-}$), and/or nitrate (NO_3^-), as indicated. Broth was inoculated with an equal mixture of the indicated strains and incubated anaerobically or under microaerobic conditions (1% O_2) for 16 h.

(A) Competitive fitness of the wild-type strain (AJB715) and a mutant lacking both FDH-N and -O activity (SW1197).

(B) Competitive fitness of the complemented Δ*fdnG phoN*::*fdnG* (RC142) mutant and a mutant lacking both FDH-N and -O activity (SW1197).

(C) Competitive fitness of the complemented Δ*fdoG phoN*::*fdoG* (RC141) mutant and a mutant lacking both FDH-N and -O activity (SW1197).

Bars represent the geometric mean ± geometric standard error. Each dot represents one biological replicate. **, $p < 0.01$; ***, $p < 0.001$.

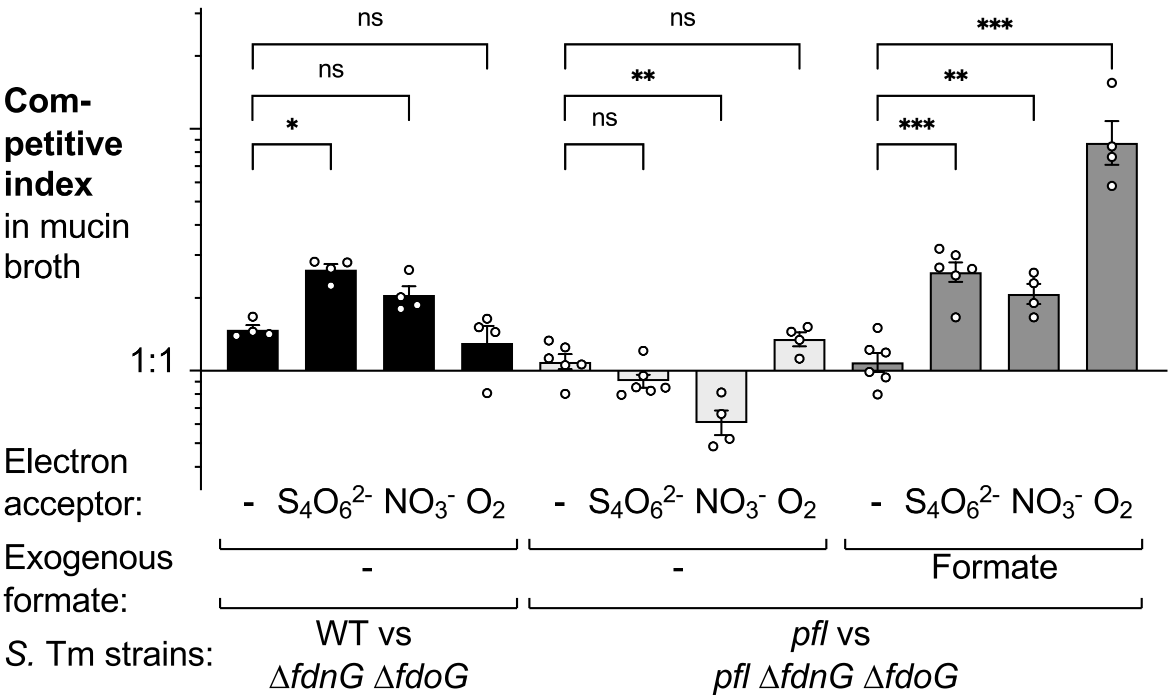

Figure S3. *S.* Tm utilizes formate produced by *S.* Tm PFL under anaerobic conditions for formate oxidation *in vitro.* Related to Fig. 4.

Mucin broth was supplemented with 4 mM of formate, tetrathionate ($S_4O_6^{2-}$), and/or nitrate (NO_3^-), as indicated. Broth was inoculated with an equal mixture of the indicated strains and incubated anaerobically or under microaerobic conditions (1% O_2) for 16 h. Bars represent the geometric mean ± geometric standard error. Each dot represents one biological replicate. *, $p < 0.05$; **, $p < 0.01$; ***, $p < 0.001$; ns, not statistically significant.
